# The Impact of Inflammatory Hyperreflective Spots and Hard Exudates on Retinal Function in Diabetic Retinal Disease

**DOI:** 10.1167/tvst.15.7.34

**Published:** 2026-07-30

**Authors:** Katharina Wall, Aaron Schumacher, Samuel A. Chang, Anna Sophia Jauch, Lukas Goerdt, Marlene Saßmannshausen, Frank G. Holz, Andreas Pollreisz, Thomas Ach

**Affiliations:** 1 Department of Ophthalmology, University Hospital Bonn, Bonn, Germany; 2 Department of Ophthalmology, Medical University Vienna, Vienna, Austria

**Keywords:** diabetic retinal disease, diabetic retinopathy, high resolution, optical coherence tomography, OCT, retinal sensitivity, microperimetry, hyperreflective spots, hard exudates, structure–function correlation

## Abstract

**Purpose:**

To characterize the topography of hyperreflective spots (HRSs) and hard exudates (HEs) and to analyze their association with localized retinal sensitivity in diabetic retinal disease (DRD).

**Methods:**

In this prospective cross-sectional study, diabetic patients (type I/II) underwent increased axial resolution optical coherence tomography (OCT) and mesopic microperimetry. HRSs (<30 µm, without back shadowing) and HEs (>30 µm, with back shadowing) were annotated in OCT scans and spatially matched to microperimetry test points across Early Treatment Diabetic Retinopathy Study (ETDRS) subfields. Eyes with confounding OCT biomarkers (disorganization of the retinal inner layers, ellipsoid zone/external limiting membrane disruption, vitreomacular traction) were excluded. Linear mixed-effects models were used to evaluate the associations between lesion types and localized retinal sensitivity.

**Results:**

Fifty-one eyes of 51 patients were included (mean age, 55.9 ± 14.8 years; diabetic retinopathy severity scale, 3.3 ± 1.6; hemoglobin A1c, 7.6% ± 2.0%). HRSs (n = 2308) were present in all study eyes, while HEs (n = 1222) were present in 21 eyes. Normalized to ETDRS subfield area, both HRSs (229.2 /mm^2^) and HEs (82.8 /mm^2^) showed the highest densities in the central subfield. Higher HRS load was independently associated with reduced retinal sensitivity (β = −0.06, *P* = 0.011), whereas no significant association was observed for HEs (*P* = 0.461); this finding should be interpreted with caution given the limited number of eyes with HEs.

**Conclusions:**

HRSs and HEs demonstrate distinct spatial distributions and functional associations in DRD, with HRSs independently linked to localized sensitivity loss.

**Translational Relevance:**

HRSs may serve as an accessible biomarker of early neuroretinal impairment and could improve patient stratification and treatment evaluation in clinical trials.

## Introduction

Diabetic retinal disease (DRD) is a progressive complication of diabetes mellitus (DM) and traditionally characterized by funduscopic microvascular abnormalities such as microaneurysms, hemorrhages, and vascular leakage on fluorescein angiography.^1^ However, current evidence suggests that neuroinflammatory processes may precede and potentially contribute to subsequent microvascular dysfunction of the neurovascular unit.^2^^–^^6^ Among the structural features visualized on optical coherence tomography (OCT), hyperreflective spots (HRSs) and hard exudates (HEs) have gained particular attention as clinically accessible biomarkers that may reflect distinct components of this neurovascular pathology.^7^^–^^10^

**Figure 1.**
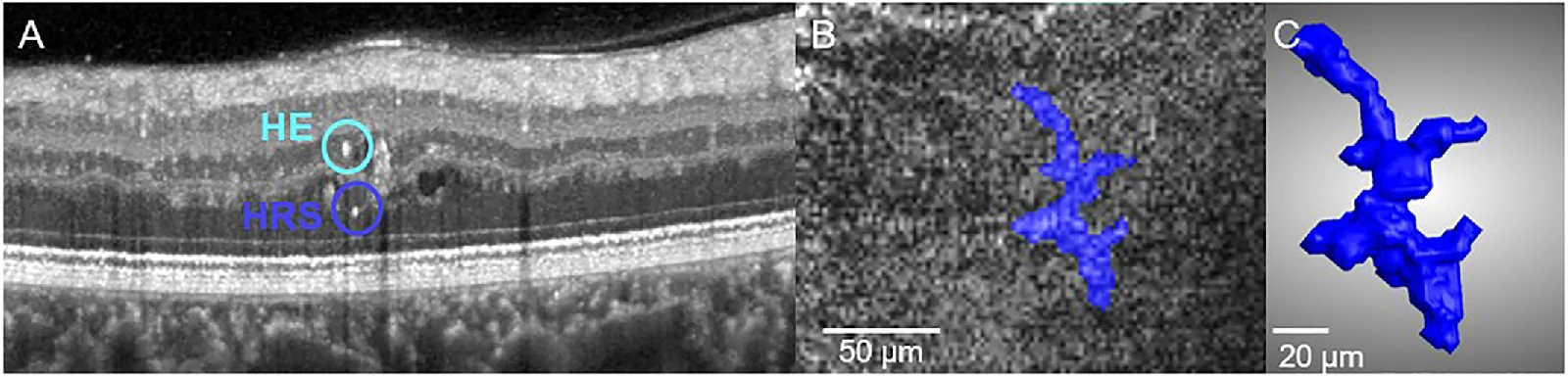
OCT/adaptive optics (AO)–OCT visualization of HRSs and HEs. (**A**) Increased axial resolution spectral-domain OCT B-scan of a 49-year-old male patient with nonproliferative diabetic retinopathy (NPDR), illustrating a representative HRS (*blue circle*) within the outer nuclear layer and an HE (*turquoise circle*) located in the outer plexiform layer. (**B**) Visualization of a single microglia-like cell (*blue*) at the level of the inner limiting membrane in the human retina of the left eye of a 39-year-old male patient with moderate NPDR. (**C**) Three-dimensional rendering of the same microglia-like cell shown in the en face image, illustrating the ramified processes extending from a central soma.

HRSs, typically identified on spectral-domain OCT (SD-OCT) as small (<30 µm), round dots with reflectivity comparable to the retinal nerve fiber layer (RNFL),^7^ are believed to represent activated microglia^11^^–^^13^ or other inflammatory by-products and are recognized as early indicators of neuroinflammation.^14^^,^^15^ HRSs are localized in the inner retina in a ramified state and gain motility as DRD develops and move toward the outer retina, as visualized by increased axial resolution OCT and adaptive optics–OCT in Figure 1.^16^^–^^18^ In contrast, HEs, defined as larger (>30 µm) extravascular aggregations of lipids and lipoproteins with back shadowing on OCT and reflectivity similar to the retinal pigment epithelium (RPE),^7^ are thought to originate from chronic vascular leakage due to breakdown of the inner blood–retinal barrier.^8^^,^^9^^,^^19^^,^^20^ HEs are often located in the outer plexiform layer along the wall of inner retinal cysts, which is recognized as the “pearl necklace sign.”^21^ While both lesion types are frequently present in DRD, their topographical distribution, relationship to disease severity, and functional consequences remain incompletely understood.

Recent advancements in OCT technology, such as the investigational High-Resolution OCT (High-Res OCT; Heidelberg Engineering, Heidelberg, Germany),^22^ have enabled imaging with substantially improved axial resolution (2.9 µm compared to 7.2 µm in standard SD-OCT) by leveraging a shorter central wavelength (from 880 to 853 nm) and an expanded spectral bandwidth (from 50 to 137 nm).^22^ In patients with age-related macular degeneration (AMD)^23^ and DRD,^24^ the increased axial resolution led to improved visualization of individual retinal layers and subtle pathologic features. The enhanced image quality may allow the reliable detection and differentiation of HRSs and HEs, including discrimination of HRSs from small capillaries or microaneurysms, and may allow for accurate quantification within individual retinal layers.^23^^–^^25^

In this study, we analyze HRSs and HEs as morphologically and spatially distinct intraretinal lesion types by evaluating their topographical and layer-specific distribution across different diabetic retinopathy severity scale (DRSS)–based DRD stages.^26^ We further examine whether the presence of HRSs and HEs within the retina is associated with clinically relevant differences in localized retinal function, thereby addressing their respective roles in functional impairment in DRD.

## Methods

### Patient Selection

This prospective cross-sectional study was conducted at the University Eye Hospital Bonn, Germany, as part of the multimodal imaging in diabetics (MIND) study (DRKS00036499) between July 2025 and December 2025. The ethics committee of the University of Bonn approved the study protocol (#2025-147-BO), and all study procedures adhered to the tenets of the Declaration of Helsinki. Before study inclusion, each patient provided written informed consent.

Inclusion criteria were age ≥18 years, previous or newly diagnosed type 1 or type 2 DM, sufficient image quality in the OCT imaging (coverage of macular area, absence of motion artifacts, adequate contrast and signal-to-noise ratio), and fixation stability (≥75% of stimuli within the 2° radius [P1 ≥75%]) in the microperimetry assessment. Exclusion criteria were the presence of other retinal diseases (e.g., AMD, macular dystrophies, retinal vascular occlusions, macular holes, or vitreomacular traction), significant media opacities (cataract or vitreous floaters), or high refractive error (>4.0 D spherical or >2.0 D cylindrical). To ensure that structure–function associations are not confounded by established markers of photoreceptor or inner retinal damage, eyes showing structural OCT biomarkers known to impair or alter microperimetry responses were excluded, including disorganization of the inner retinal layers (DRIL); disruption, attenuation, or discontinuity of the ellipsoid zone (EZ) or the external limiting membrane (ELM); vitreomacular traction; and the presence of subretinal fluid. Eyes with intraretinal fluid consistent with diabetic macular edema (DME) or intraretinal cysts (IRCs) in at least one Early Treatment Diabetic Retinopathy Study (ETDRS) subfield were included. If both eyes were eligible, the eye with the better best-corrected visual acuity (BCVA) was included in the analysis. A total of 111 eyes were screened for eligibility. Of these, 60 eyes were excluded due to the predefined OCT-based criteria, including DRIL, disruption of the EZ or ELM, vitreomacular traction, or the presence of subretinal fluid (Supplementary Fig. S1). The final analysis included 51 eyes.

Patient self-reporting was used to collect medical information, which included the specific type of DM, duration since initial diabetes diagnosis, current hemoglobin A1c (HbA1c) level, and current DM therapy.

### Clinical Examination and Image Acquisition

 All participants underwent BCVA testing, biomicroscopy of the anterior segment, and indirect funduscopy (after pupil dilation with 1.0% tropicamide and 2.5% phenylephrine) prior to retinal imaging. Severity of DRD was graded based on the international clinical DRSS.^26^ The retinal imaging protocol included High-Res OCT (30° × 25°, ART 25, 121 B-scans; axial resolution: 2.9 µm, wavelength 853 nm, power 2.2 mW; Heidelberg Engineering) and color fundus photography (131° Clarus; Carl Zeiss Meditec AG, Jena, Germany).

### Retinal Sensitivity Assessment

The S-MAIA device (CenterVue/iCare, Oy, Finland) was used to assess mesopic retinal sensitivity. The test configuration included 68 stimuli within the central 24° (6 mm) of the retina covering all ETDRS subfields (4-2 dB staircase strategy, Goldmann III stimulus size of 0.43°, background luminance of 4 apostilb [1.3 cd/m^2^], 36 dB dynamic testing range, fixation tracking speed set at 25 Hz). Retinal sensitivity for each ETDRS subfield was calculated as the mean threshold of all test points in that region.

### Image Analysis

Volumetric High-Res OCT imaging data were automatically segmented using the device's internal software (Spectralis Viewer Module 6.3.2.0; Eye Explorer, Heidelberg Engineering), reviewed in each of the 121 B-scans, and manually corrected, if necessary, based on the anatomic landmarks as proposed by Staurenghi et al.^27^ Central subfield thickness (CST) was extracted from the device's internal “Thickness Map” tool. Subsequently, the corrected segmentation files (XML format) were then exported from the Spectralis Viewer and imported into ImageJ^28^ (National Institutes of Health, Bethesda, MD, USA) for quantitative analysis.

**Figure 2.**
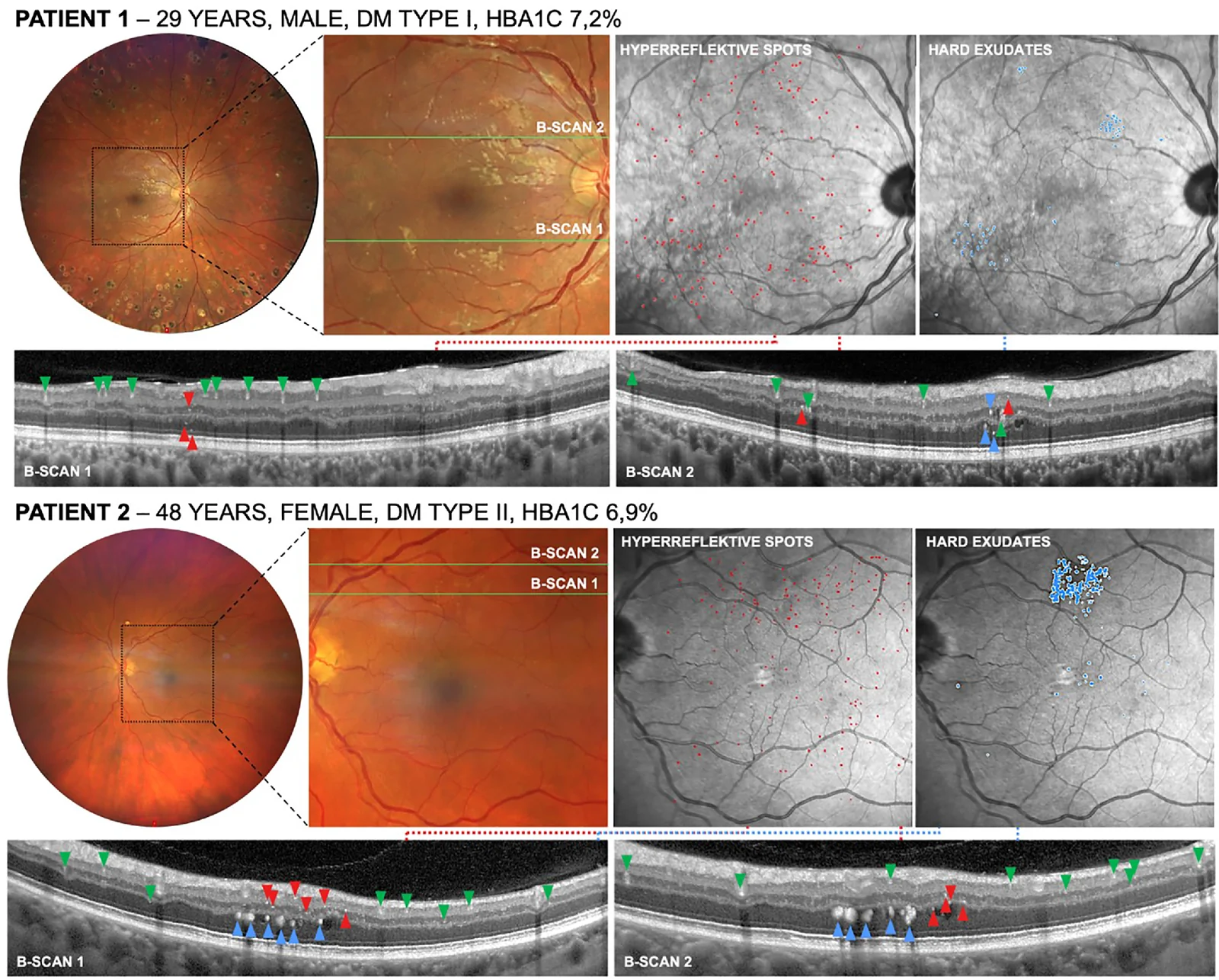
Representative multimodal imaging and mapping of HRSs and HEs in High-Resolution OCT B-scans. High-resolution OCT B-scans (axial resolution 2.9 µm) were used to identify and classify HRSs and HEs based on predefined morphologic criteria. HRSs were defined as small (<30 µm), punctate, round lesions with reflectivity similar to the RNFL and without back shadowing. HEs were defined as larger (>30 µm) hyperreflective deposits with reflectivity similar to the RPE and back shadowing. All lesions were manually annotated on the corresponding B-scans. Using the Mark HRF OCT ImageJ plugin, the lesion coordinates were automatically transferred to matched en face projections, enabling precise topographic visualization of HRS (*red*) and exudates (*cyan*) across the macula for each patient. For illustrative purposes, small retinal capillaries were additionally marked in *green*. These structures were characterized by linear or tubular morphology and back shadowing. They were not classified as HRS or HE, nor included in any quantitative analysis.

HRSs (referred to as “I-HRF” in the publication by Frizziero et al.^7^) were defined as intraretinal punctate lesions smaller than 30 µm extension with reflectivity comparable to the RNFL and without associated back shadowing. In contrast, HEs (referred to as “E-HRF” in the publication by Frizziero et al.^7^) were defined as hyperreflective lesions >30 µm in width with a reflectivity similar to the RPE and with back shadowing. Annotation was performed manually on each B-scan using the “Mark HRF OCT” plugin in ImageJ^28^ (freely available for download at https://sites.imagej.net/CreativeComputation/), which records the size, coordinates, and intraretinal location of each lesion (Fig. 2). In regions of dense vascularization, capillaries were identified based on their linear morphology and continuity across adjacent B-scans and were not annotated as HRSs, whereas HRSs appeared as isolated, noncontiguous structures. Lesions demonstrating reflectivity patterns at luminal interfaces were classified as microaneurysms. Hyperreflective signals lacking clearly defined borders or consistent morphology were not annotated. Speckle noise was identified based on its random, nonreproducible appearance across consecutive scans and was excluded from annotation. Structures not fulfilling predefined criteria were not annotated. A subset of 10 eyes was regraded (by authors KW and AS) to assess interreader reliability using the intraclass correlation coefficient (ICC). Graders were masked to patients’ identities and all clinical data during annotation. After annotation, the recorded lesion coordinates were projected onto corresponding en face images to map the topographical distribution of HRSs and HEs across standardized ETDRS subfields. Their frequency within the following individual retinal layers ganglion cell layer (GCL), inner plexiform layer (IPL), inner nuclear layer (INL), outer plexiform layer (OPL), and outer nuclear layer (ONL) was also recorded. IRCs were identified on each B-scan as hyporeflective, well-demarcated intraretinal cavities and were manually annotated in ImageJ in an analogous manner. Diffuse retinal thickening without clearly demarcated cystoid spaces was not classified as IRCs. No minimum size threshold was applied, and grading was independent of CST. The spatial coordinates of IRCs were likewise projected onto corresponding en face images to generate subfield-based density maps.

### Statistical Analysis

All statistical analyses were conducted using SPSS (IBM SPSS Statistics, Armonk, NY, USA, version 27). Descriptive statistics were calculated for all demographic, clinical, and imaging variables. The topographical distribution of HRSs and HEs across ETDRS subfields was summarized using absolute counts and relative proportions.

To evaluate whether the distribution of HRSs and HEs differed across DRSS levels, lesion counts were analyzed separately for each retinal layer (GCL, IPL, INL, OPL, ONL). The data were nonnormally distributed (Shapiro–Wilk test), and therefore, comparisons across DRSS categories were performed using the Kruskal–Wallis test.

To assess structure–function relationships, linear mixed-effect models were used with local retinal sensitivity (per ETDRS subfield) as the dependent variable. Independent variables included HRS count per ETDRS subfield, HE count per ETDRS subfield, age, diabetes duration, the presence of IRCs, DRSS stage, spatially matched subfield thickness, intravitreal therapy (yes/no), and prior panretinal photocoagulation (yes/no), with patient ID as a random intercept. Regression coefficients (β), 95% confidence intervals (CIs), and *P* values were reported. Statistical significance was considered at *P* < 0.05, two-sided.

## Results

A total of 51 eyes from 51 patients with DM were included in the study (mean age 55.9 ± 14.8 years; 23 females [45.1%]). Mean duration of diabetes was 20.0 ± 11.9 years, and mean HbA1c was 7.6% ± 2.0%. Twenty patients (39.2%) had type 1 DM and 31 (60.8%) had type 2 DM, and mean body mass index (BMI) was 29.9 ± 6.5 kg/m^2^. Mean BCVA was 0.10 ± 0.14 logarithm of the minimum angle of resolution (logMAR). Detailed characteristics of the cohort are summarized in Table 1.

**Table 1.** Study Population's Characteristics

Variable	*P* Value
Cohort characteristics	
Participants, *n*	51
Male, *n* (%)	28 (54.9)
Female, *n* (%)	23 (45.1)
Age (years), mean ± SD	55.9 ± 14.8
Type of diabetes	
Type 1, *n* (%)	20 (39.2)
Type 2, *n* (%)	31 (60.8)
Duration of diabetes (years), mean ± SD	20 ± 11.9
HbA1c (%), mean ± SD	7.6 ± 2
BMI (kg/m^2^), mean ± SD	29.9 ± 6.5
Ocular characteristics	
BCVA (logMAR), mean ± SD	0.1 ± 0.1
Eyes, *n*	51
Right, *n* (%)	25 (49)
Left, *n* (%)	26 (51)
Lens status	
Phakic, *n* (%)	31 (60.8)
Pseudophakic, *n* (%)	20 (39.2)
DRSS, mean ± SD	3.3 ± 1.6
DRSS 1, *n* (%)	13 (25.5)
DRSS 2, *n* (%)	5 (9.8)
DRSS 3, *n* (%)	6 (11.8)
DRSS 4, *n* (%)	5 (9.8)
DRSS 5, *n* (%)	22 (43.1)
Current presence of intraretinal cysts, *n* (%)	19 (37.3)
Central subfield thickness (µm)	293 ± 36.4
Treatment in the study eye	
Current intravitreal anti-VEGF/steroid therapy, *n* (%)	15 (29.4)
Previous panretinal laser photocoagulation, *n* (%)	21 (41.2)

BMI, body mass index; logMAR, logarithm of the minimum angle of resolution.

All 51 eyes had HRSs, whereas additional HEs were present in 21 eyes. Among the eyes with HEs, 19 eyes (90.5%) showed IRCs on OCT. Mean CST in all patients was 293 ± 36.4 µm. Overall, 2308 HRSs and 1222 HEs were identified. ICC for HRS and HE count between the two graders was 0.88.

### Retinal Layer-Specific Distribution of HRSs and HEs

HRSs and HEs were present in all inner retinal layers (Table 2). HRSs were most frequently located in the INL (31.9%), ONL (29.4%), and OPL (16.7%), while HEs were predominantly found in the OPL (37.9%) and ONL (38.3%), with only minimal counts in inner retinal layers.

**Table 2.** HRS and HE Count Per Retinal Layer

	HRS		HE	
Retinal Layer	*n*	%	*n*	%
Ganglion cell layer (GCL)	235	10.3	8	0.7
Inner plexiform layer (IPL)	269	11.8	65	5.3
Inner nuclear layer (INL)	730	31.9	217	17.8
Outer plexiform layer (OPL)	382	16.7	463	37.9
Outer nuclear layer (ONL)	672	29.4	467	38.3

Counts and percentage distribution of HRSs and HEs within individual retinal layers (GCL, IPL, INL, OPL, ONL). HRSs predominantly localize to the inner nuclear layer, whereas HEs accumulate mainly within the OPL and ONL.

**Figure 3.**
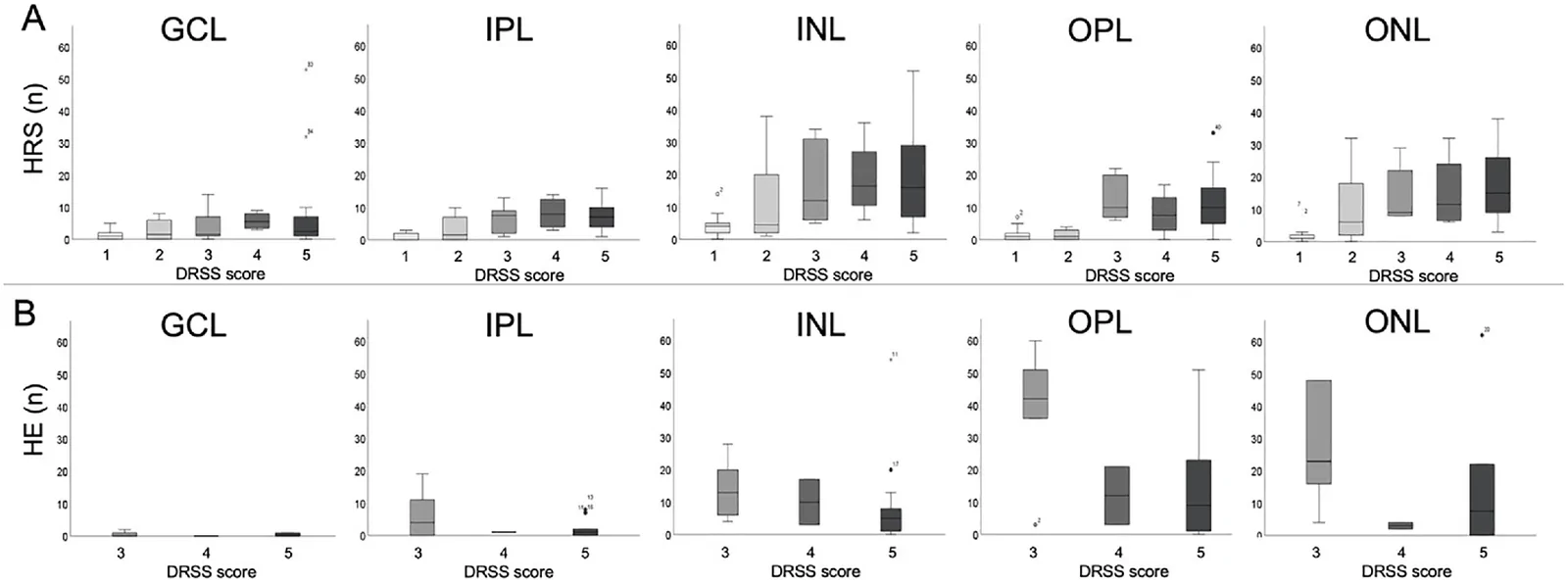
Layer-specific distribution of HRSs (**A**) and HEs (**B**) across DRSS severity levels. (**A**) Boxplots showing the distribution of the number of HRSs per eye across individual retinal layers (GCL, IPL, INL, OPL, ONL) across increasing DRSS severity levels. Higher DRSS stages exhibit a clear accumulation of HRSs, particularly within the INL, OPL, and ONL. (**B**) Boxplots illustrating the distribution of the number of HEs per eye across retinal layers. HEs are predominantly located in the OPL and ONL and occur in our cohort exclusively in eyes with moderate to severe diabetic retinopathy (DRSS ≥3).

HRS counts differed significantly across DRSS stages in the IPL (*P* < 0.001), INL (*P* = 0.002), OPL (*P* < 0.001), and ONL (*P* < 0.001), whereas no significant group differences were observed for the GCL (*P* = 0.109). In general, HRS counts were higher in more advanced DRSS stages. HEs occurred exclusively in eyes with DRSS ≥3. Among these eyes, HE counts did not differ significantly between DRSS stages in any retinal layers (all *P* > 0.17) and appeared largely independent of disease severity. The distribution of HRSs and HEs across DRSS severity levels for each retinal layer is shown in Figure 3. Corresponding rank distributions and Kruskal–Wallis statistics are provided in the Supplementary Tables S1 and S2.

### Topographical Distribution of HRSs, HEs, and IRCs

HRSs were present in all ETDRS subfields. HRS density was highest in the central subfield (229.2 /mm^2^), followed by the outer temporal subfield (106.4 /mm^2^), as well as the subfields of the inner ring (94.2–97.4 /mm^2^), and was lowest in the outer nasal subfield (39.0 /mm^2^). HE densities were highest in the central subfield (82.8 /mm^2^), followed by the outer temporal (73.0 /mm^2^) and inner temporal (59.8 /mm^2^) subfields, as well as the inner superior (65.6 /mm^2^) and outer superior (53.2 /mm^2^) subfields, with progressively lower densities toward nasal regions, reaching lowest values in the outer nasal subfield (11.5 /mm^2^).

IRCs were present in 21 of 51 eyes (41.2%). On a subfield level, IRCs were most frequently observed in the outer temporal subfield (67.9 /mm^2^), the central subfield (54.4 /mm^2^), the inner temporal subfield (59.9 /mm^2^), and the outer superior subfield (45.1 /mm^2^).

**Figure 4.**
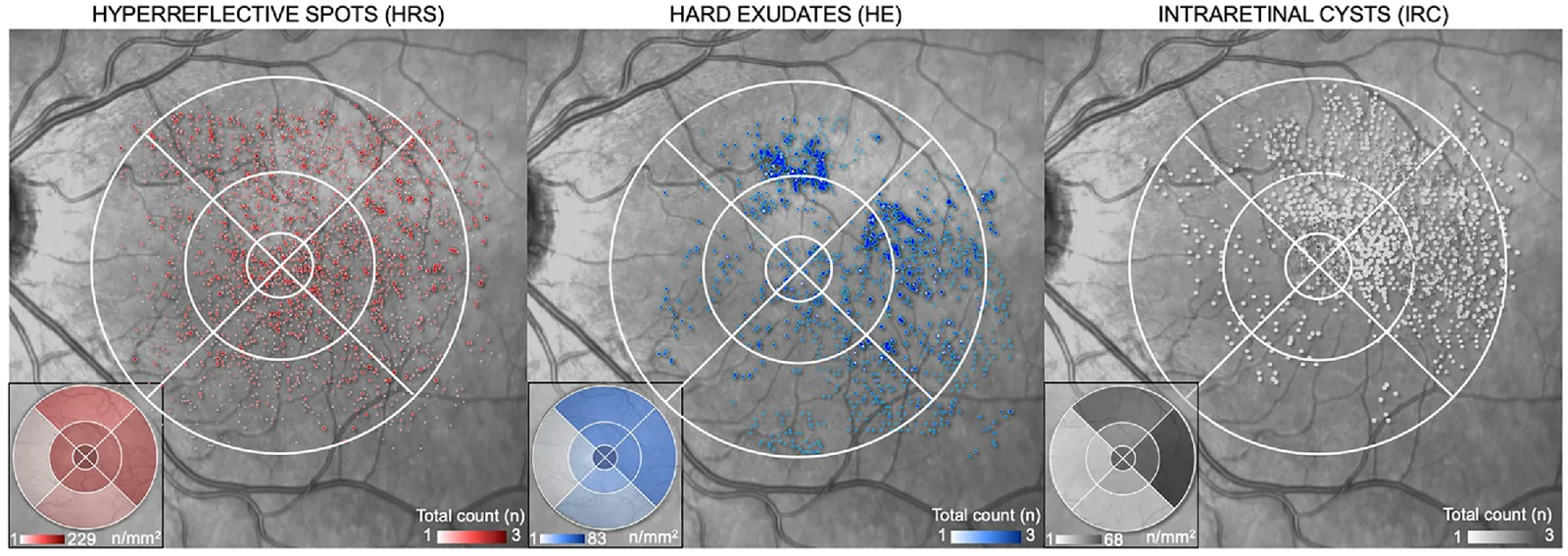
Spatial distribution and regional density of HRSs, HEs, and IRCs. *Left*: En face projection illustrating the pointwise spatial distribution of all HRSs (*red*) overlaid on a standardized near-infrared fundus image. Each dot represents the location of an individual lesion detected on high-resolution OCT. Color intensity reflects the degree of overlap across the stack (scale 1–3), with lighter colors indicating less overlap. The inset shows the ETDRS subfield-based density map (number per mm^2^), normalized to subfield area. *Middle*: En face projection illustrating the spatial distribution of all HEs (*blue*) using the same conventions. The *inset* displays the corresponding ETDRS subfield-based density map (number per mm^2^). *Right*: En face projection illustrating the spatial distribution of all IRCs (*white/gray*) across the macula using the same conventions. Each dot represents the location of an intraretinal cyst identified on OCT. Dot size does not reflect cyst size; only the presence and spatial location of cysts are illustrated. The *inset* shows the ETDRS subfield-based density map (number per mm^2^), normalized to subfield area.

HEs were most commonly detected in ETDRS subfields, with a higher prevalence of IRCs, particularly in the temporal and superior subfields. In general, HE counts were significantly higher in eyes with IRCs compared to eyes without IRCs (Mann–Whitney *U* = 33.0, *z* = −5.49, *P* < 0.001). The spatial distributions of HRS, HE and IRC prevalence across ETDRS subfields are presented in Table 3 and visualized in Figure 4.

**Table 3.** Distribution of HRSs, HEs, and IRCs Across ETDRS Subfields

	HRS		HE		IRC	
ETDRS Subfield	*n*	/mm^2^	*n*	/mm^2^	*n*	/mm^2^
Central subfield	180	229.2	65	82.8	43	54.4
Inner ring—superior	152	96.8	103	65.6	55	35.0
Inner ring—temporal	153	97.4	94	59.8	94	59.9
Inner ring—inferior	151	96.1	59	37.6	31	19.7
Inner ring—nasal	148	94.2	41	26.1	19	12.1
Outer ring—superior	445	83.9	282	53.2	239	45.1
Outer ring—temporal	564	106.4	387	73.0	360	67.9
Outer ring—inferior	308	58.1	130	24.5	80	15.1
Outer ring—nasal	207	39.0	61	11.5	42	7.9

Absolute counts and area-normalized densities (/mm^2^) of HRSs, HEs, and IRCs of each ETDRS subfield (central, inner ring, outer ring). Lesion densities (/mm^2^) were calculated by normalizing absolute lesion counts to the anatomic area of each ETDRS subfield (central subfield: radius 0.5 mm; area 0.79 mm^2^; inner ring: radius 0.5–1.5 mm; area 6.28 mm^2^ corresponding to 1.57 mm^2^ per subfield; outer ring: radius 1.5–3 mm; area 21.21 mm^2^ corresponding to 5.30 mm^2^ per subfield).

### Association of HRSs and HEs With Localized Retinal Sensitivity

Retinal sensitivity was analyzed for each ETDRS subfield. In the multivariable linear mixed-effect model (Table 4), higher HRS count was significantly associated with lower retinal sensitivity (β = −0.062; 95% confidence interval [CI], −0.110 to −0.015; *P* = 0.011). Based on the observed distribution of HRS counts, the difference between the 25th and 75th percentiles corresponded to 6 HRSs per subfield, translating into an estimated difference in retinal sensitivity of approximately 0.37 dB. In contrast, HE count was not significantly associated with retinal sensitivity (*P* = 0.461). Among the additional covariates, DRSS stage (β = 0.718, *P* = 0.032) and ETDRS subfield (β = −0.174, *P* = 0.016) were significantly associated with retinal sensitivity. Spatially matched subfield thickness, treatment variables (intravitreal therapy and prior panretinal photocoagulation), age, diabetes duration, and the presence of IRCs were not significant predictors (all *P* > 0.18).

**Table 4.** Multivariable Linear Regression Models for Local Retinal Sensitivity

Predictor	β	95% CI	*P* Value
HRS count (per subfield)	**−0.062**	**−0.110 to −0.015**	**0.011**
HE count (per subfield)	0.012	−0.020 to 0.044	0.461
Age	−0.024	−0.058 to −0.011	0.182
DRSS	**0.718**	**0.062 to 1.375**	**0.032**
DM duration	−0.014	−0.041 to 0.013	0.295
Presence of IRC (per subfield)	−0.152	−0.935 to 0.632	0.702
ETDRS subfield	**−0.174**	**−0.315 to −0.033**	**0.016**
Spatially matched subfield thickness	−0.001	−0.002 to 0.001	0.227
Previous or current anti-VEGF/intravitreal steroid therapy	−0.843	−2.201 to 0.516	0.222
Previous panretinal photocoagulation	−0.249	−1.285 to 0.787	0.635

Results of a multivariable linear regression model examining the association between HRS count, HE count, age, diabetic retinopathy severity (DRSS), diabetes duration, presence of IRCs, retinal location (ETDRS subfield), spatially matched subfield thickness, and treatment variables (intravitreal therapy and prior panretinal photocoagulation) with retinal sensitivity (dependent variable). Estimates are presented as regression coefficients (β), 95% confidence intervals (CIs), and *P* values.

Bold values indicate statistically significant results (*P* < 0.05).

## Discussion

In this study, we characterized HRSs and HEs as two biologically distinct intraretinal lesion types in DRD by examining their layer-specific locations, distribution across the ETDRS grid, and their impact on retinal function. HRSs were present in all eyes and were more prevalent in more advanced DRSS stages, whereas HEs appeared only in a subset of patients and were associated with the presence of IRC. Importantly, HRS load was independently associated with reduced localized retinal sensitivity, while the presence of HEs showed no significant functional effect, underscoring potential divergent pathogenic roles.

### Retinal Layer-Specific Distribution of HRSs and HEs

HRSs and HEs exhibited distinct intraretinal layer distributions, reflecting their different biological origins. HRSs were present in all retinal layers, with a relatively greater proportion in the nuclear layers and the OPL.^29^ This pattern is consistent with the spatial dynamics of retinal microglia, the resident innate immune cells of the retina.^30^ While HRSs are widely interpreted in the literature as representing activated microglial cells, their exact biological correlate remains uncertain, and alternative explanations such as inflammatory cells, lipid aggregates, or migrating RPE cells have been proposed. Histologic^31^ and experimental studies^32^^,^^33^ demonstrated that retinal microglia preferentially localize to the GCL and IPL and migrate toward the outer retina under pathologic conditions, including diabetes. Microglia originate from yolk sac–derived myeloid progenitors and normally reside as ramified cells within the inner retinal layers.^34^ In a physiological environment, microglia continuously monitor synapses, maintain neuronal homeostasis, and scan their microenvironment with dynamic processes.^35^^,^^36^ In DRD, chronic hyperglycemia, oxidative stress, and advanced glycation end products induce early microglial activation characterized by NF-κB signaling,^37^ cytokine production, retraction of fine cellular projections, and transition toward an amoeboid morphology.^29^^,^^38^^,^^39^ Activated microglia subsequently redistribute into deeper retinal layers (INL, OPL, ONL), where they accumulate around microaneurysms, ischemic areas, and zones of photoreceptor stress.^12^^,^^15^ This transition from inner retinal surveillance to clusters in deeper retinal layers aligns with the increased INL/ONL HRS burden in eyes with more advanced disease in our cohort.

HEs, in contrast, were almost exclusively located within the OPL and ONL, in agreement with histologic^15^^,^^40^ and clinical OCT studies,^9^^,^^41^ demonstrating that they represent extracellular precipitates of lipids, lipoproteins, and proteinaceous material (fibrinogen, albumin, immunoglobulins) that accumulate where chronic vascular leakage and fluid pooling occur in DME.^9^^,^^42^^,^^43^ Although the retinal vasculature is also present within the inner retinal layers, its preferential localization to the outer retina reflects the architecture of Henle's fiber layer^44^ and the directionality of interstitial fluid movement, which favors centrifugal redistribution toward the OPL/ONL.^45^ Mechanistically, inflammation-induced vascular endothelial growth factor (VEGF)–mediated vascular permeability,^46^ leukocyte adhesion,^47^ and tight junction loss,^48^ particularly within the deep capillary plexus,^49^^,^^50^ promote extravasation of plasma proteins with high oncotic load. These macromolecules are subsequently redistributed and retained within the outer retinal compartments, where resorption of extravasated fluid facilitates precipitation of lipid-rich material.^45^ This explains the clustering of exudates adjacent to leakage zones despite the presence of retinal vessels in the inner retina.

Together, these observations underscore that HRSs and HEs represent biologically distinct retinal lesions: HRSs may reflect neuroinflammatory processes potentially involving microglial activation, whereas HEs represent lipid-rich extracellular deposits arising from chronic vascular leakage. This reinforces the need to analyze them separately when studying structural and functional correlates of DRD.

### Topographical Distribution Across the Macula

When considering absolute lesion counts, HRSs were most frequent in the ETDRS outer ring, but after normalization to the subfield area, HRS density was highest in the central ETDRS subfield. Although precise quantitative data comparing microglial density across ETDRS subfields are not available, microglia preferentially inhabit perivascular regions outside the foveal avascular zone.^51^ A primate histologic study demonstrates that microglial density is lowest in the foveal center and increases with retinal eccentricity, with a relative enrichment in the vascularized regions approximately 1 to 3 mm from the foveal center.^52^ Experimental models (ex vivo human retina and mouse model) of DRD further demonstrate that microglial activation is spatially associated with capillary dropout, oxidative stress, and local tissue hypoxia,^53^ changes that often occur in mid-macular zones corresponding to the inner and outer ETDRS ring.^15^ In contrast to this physiological distribution, we observed a comparatively high density of HRSs in the central ETDRS subfield. This may reflect disease-related alterations of the foveal microenvironment in DRD. Histologic studies in human DRD demonstrate that activated microglia increasingly cluster around ischemic regions, microaneurysms, and sites of neurovascular unit disruption.^15^^,^^29^^,^^53^ In eyes with DME,^15^ local metabolic stress and hypoxia have been implicated in microglial recruitment in DRD and may contribute to the elevated central HRS density in our cohort.

HE density was highest in the central ETDRS subfield (82.8 /mm^2^), which indicates a high local burden of lipid deposition. Importantly, this high central HE density coincided with the presence of IRCs (54.4 /mm^2^ in the central subfield), supporting a close spatial relationship between HE accumulation and intraretinal fluid. Notably, mean CST remained below 300 µm in the overall cohort, indicating that the observed IRC reflected mild macular edema rather than advanced foveal thickening. Subfields with the highest IRC prevalence in our cohort, particularly the outer temporal (67.9 /mm^2^) and inner temporal (59.9 /mm^2^) subfields, also showed high HE densities (73.0 /mm^2^ and 59.4 /mm^2^, respectively). Additionally, HEs were significantly less frequent in nasal ETDRS subfields. The nasal macula is known to exhibit relatively preserved perfusion and less capillary dropout,^54^^,^^55^ reducing the likelihood of chronic leakage. Furthermore, macular fluid redistribution follows structural gradients influenced by Henle fiber orientation and Müller cell architecture,^44^^,^^56^ which favor temporal and superior accumulation. It should also be considered that prior or current anti-VEGF therapy may have modulated vascular permeability and retinal thickness. Anti-VEGF treatment can reduce intraretinal fluid while lipid deposition persists, potentially influencing the spatial relationship between HE density and IRC distribution in our cohort. Future longitudinal studies incorporating DME duration and treatment history are required to better delineate these effects.

### Structure–Function Correlation

HRS load was associated with lower localized retinal sensitivity, whereas HE counts did not show a significant functional association. This finding is consistent with evidence of early neuroretinal dysfunction in DRD as a primary pathophysiological disease component.^57^ Neurodegenerative processes include chronic low-grade inflammation,^58^ glutamate excitotoxicity,^59^ oxidative stress,^60^ mitochondrial dysfunction,^61^ impaired neurotrophic signaling,^62^ and microglia-driven synaptic remodeling.^15^ These processes collectively compromise neuronal survival and synaptic integrity in the absence of overt vascular lesions. Functional impairments such as reduced retinal sensitivity,^63^^,^^64^ contrast sensitivity loss,^65^ and electrophysiologic abnormalities^66^ occur in eyes without clinical retinopathy, underscoring the early vulnerability of retinal neurons.

Within this framework, HRSs likely represent localized neuroinflammatory activity, and their colocalization with regions of reduced sensitivity may reflect underlying neuroretinal dysfunction rather than a direct structural effect. The persistence of this association after adjustment for spatially matched subfield thickness suggests that the observed functional impairment is not solely driven by retinal thickening or edema, although this relation has been demonstrated before.^67^ Similarly, the lack of association with treatment variables indicates that prior therapeutic interventions are unlikely to account for this relationship. However, residual confounding by treatment effects cannot be entirely excluded. From a clinical perspective, although the effect size is small, the consistent association between HRSs and retinal sensitivity suggests that HRSs may serve as a marker of localized neuroretinal dysfunction. When considered relative to the test–retest variability of microperimetry (∼2–3 dB), the effect is likely below perceptibility at the individual level but may be relevant at the group level.

In contrast, the presence of HEs was not associated with significantly reduced retinal sensitivity in our cohort, which aligns with previous work showing that HEs do not necessarily correspond to neuronal dysfunction unless accompanied by disruption of the EZ/ELM or extensive edema.^68^ However, this finding should be interpreted with caution given the limited number of eyes with HEs, which may have reduced statistical power to detect more subtle functional effects. It should therefore be considered a preliminary observation rather than definitive evidence of absence of effect.

Prior studies have reported associations between hyperreflective lesions and visual function or treatment response in DME, including correlations with microperimetric sensitivity, fixation stability, or visual acuity.^8^^,^^68^^–^^71^ However, a limitation of this literature is the heterogeneous use of the term “hyperreflective foci,” which often encompasses both small, nonshadowing HRSs and larger, shadowing lipid exudates. These lesion types likely represent different biological processes, and therefore, functional associations reported in earlier work may reflect mixed contributions.

Taken together, these observations highlight a distinction between the two lesion types: HRSs appear to mark areas of underlying neuroretinal dysfunction, whereas exudates in structurally preserved retina primarily reflect chronic vascular leakage without an additional, measurable functional impact.

### Limitations and Strengths

One strength of this study is the use of an increased axial resolution OCT, which allows a reliable differentiation of small hyperreflective lesions. On conventional SD-OCT, distinguishing HRSs from thin retinal capillaries, microaneurysms, or very small exudative deposits can be challenging, particularly when back shadowing is subtle or partially obscured by noise. The higher axial resolution enabled clear visualization of lesion borders, assessment of back shadowing, and thus more confident classification of HRSs versus HEs versus microvascular structures. Nevertheless, even with increased axial resolution OCT, in a small subset of hyperreflective lesions, it remains difficult to distinguish HRSs from fine capillaries, particularly in areas of dense vascularization. Excluding structural confounders such as DRIL and outer retinal band disruption allowed for a more specific functional evaluation. Furthermore, integrating spatially registered microperimetry with layer-resolved lesion mapping provided detailed structure–function correlations of both lesion types individually.

However, several limitations should also be acknowledged. First, the cross-sectional design does not permit conclusions about the temporal dynamics or causality between lesion formation and functional impairment. Second, although HRSs were present in all eyes, HEs occurred in only a subset of patients (*n* = 21). This limited the statistical power to detect more subtle functional associations with HE burden. Additionally, the applied exclusion criteria, particularly the removal of eyes with DRIL, outer retinal disruption, and other structural abnormalities, may limit the generalizability of our findings to the broader DRD population. Third, lesion annotation was performed manually, which, despite high interreader reliability, may introduce variability. Finally, microperimetry is subject to subfield-dependent normative variation and intraindividual variability, which may reduce the accuracy of sensitivity estimates. Inclusion of an age-matched control cohort in future studies would also strengthen the interpretation of localized functional alterations. Despite these limitations, combining high-resolution structural imaging, strict biomarker control, and localized functional testing offers a robust approach to understanding the differential roles of HRSs and HEs in DRD.

## Conclusions

Using increased axial resolution OCT, we distinguished HRSs and HEs as separate intraretinal biomarkers with characteristic layer-specific and topographical patterns. HRSs are independently associated with reduced retinal sensitivity, indicating localized neuroretinal dysfunction. HEs showed no functional association in structurally preserved retina. These findings highlight the importance of analyzing hyperreflective lesion subtypes separately when assessing structural and functional alterations, especially in DRD treatment trials.

## Supplementary Material

Supplement 1

Supplement 2

Supplement 3
